# HomeoboxC6 promotes metastasis by orchestrating the DKK1/Wnt/β-catenin axis in right-sided colon cancer

**DOI:** 10.1038/s41419-021-03630-x

**Published:** 2021-04-01

**Authors:** Lina Qi, Jiani Chen, Biting Zhou, Kailun Xu, Kailai Wang, Zhihao Fang, Yingkuan Shao, Ying Yuan, Shu Zheng, Wangxiong Hu

**Affiliations:** 1 Cancer Institute, Key Laboratory of Cancer Prevention and Intervention, Ministry of Education, The Second Affiliated Hospital, Zhejiang University School of Medicine, Hangzhou, Zhejiang China; 2 Department of Medical Oncology, Key Laboratory of Cancer Prevention and Intervention, Ministry of Education, The Second Affiliated Hospital, Zhejiang University School of Medicine, Hangzhou, Zhejiang China

**Keywords:** Colon cancer, Growth factor signalling

## Abstract

Patients with right-sided colon cancer (RCC) generally have a poorer prognosis than those with left-sided colon cancer (LCC). We previously found that homeobox C6 (*HOXC6*) was the most significantly upregulated gene in RCC compared to LCC. However, it remains unclear whether HOXC6 plays a role in tumor proliferation and metastasis. Our study aimed to explore the potential oncogenic role and the detailed molecular mechanism of HOXC6 in RCC. In this study, HOXC6 was validated to be overexpressed in RCC and associated with poor prognosis. Furthermore, overexpression of HOXC6 promoted the migration and invasion of colon cancer cells through inducing EMT by activating the Wnt/β-catenin signaling pathway and inhibition of DKK1 secretion. Lastly, we preliminary explored the translational effect of HOXC6 and found that silencing of HOXC6 made HCT116 and HT29 cells more sensitive to irinotecan.

## Introduction

Colorectal cancer (CRC) is one of the most common digestive tract malignant tumors, and its incidence rate and mortality rate rank third among all malignant tumors worldwide^1^. In recent years, an increasing number of studies have revealed that left- and right-sided colon cancer (LCC and RCC) differ greatly in embryology, epidemiology, molecular characteristics, and clinical manifestations^2,3^. Moreover, the different locations of CRC have large differences in prognosis and response to clinical treatment^4,5^. A recent research including 66 studies covering 1,437,846 patients showed a lower risk of death in patients with LCC than RCC (HR, 0.82; 95% CI, 0.79–0.84; *P* < 0.001), and the result was independent of stage, race, adjuvant chemotherapy, year of study, number of participants, and quality of the included studies^6^. However, the underlying molecular difference between LCC and RCC patients that may better explain the worse survival of RCC remains poorly understood.

In our previous study, we identified *HOXC6* as the most significantly upregulated gene in RCC compared to LCC in both TCGA and GSE14333 datasets, indicating that *HOXC6* may be an oncogene in RCC^7^. *HOXC6*, known as a member of the homeobox family, not only participates in vertebrate embryonic development^8^, but is also upregulated and plays vital roles in various cancer types, such as breast cancer^9^, lung cancer^10^, prostate cancer^11^, and leukemia^12^. Nevertheless, the concrete role of *HOXC6* in RCC remains unknown, and whether HOXC6 may serve as a target in RCC to improve patient survival should be urgently clarified.

In this study, we confirmed that HOXC6 was overexpressed in RCC and associated with a worse outcome. The regulatory axis HOXC6/DKK1/β-catenin contributing to metastasis via EMT was identified in RCC. Furthermore, we preliminary tested the effect of HOXC6 on the sensitivity of chemotherapy drugs and found that silencing of HOXC6 confers HCT116 and HT29 cell lines more sensitive to irinotecan. Our findings helped to better understand the heterogeneity within CRC and offered a good candidate target in RCC treatment.

## Materials and methods

### Bioinformatic analysis of TCGA and GEO datasets

All Level 3 CRC RNASeqV2 mRNA expression profiles were obtained from TCGA (08/26, 2017). The raw CEL files of GSE39582 (Affymetrix HG U133 Plus 2.0 arrays) were downloaded from Gene Expression Omnibus (GEO) and processed using the *affy* package of BioConductor. Then, the MAS5 algorithm was used for background correction, normalization, and summarization of single probes for all probe sets. The identification of differentially expressed genes (DEGs) between subgroups was performed as in our previous studies^7,13^. Significant DEGs were selected according to a false discovery rate (FDR) adjusted *P-*value < 0.05 and fold change > 2. Hallmark gene sets from the molecular signatures database (MSigDB)^14^ were used to determine whether any signatures were enriched in specific groups by gene set enrichment analysis (GSEA)^15^. Significantly enriched hallmarks were chosen according to a *P*-value < 0.05.

Survival differences between the HOXC6+ and HOXC6− groups were tested by the Kaplan–Meier method and analyzed with the log-rank test using the functions *survfit* and *survdiff* in the *survival* package in R^16^. Multivariate Cox regression was performed using the *coxph* function.

### HOXC6 immunohistochemistry (IHC) for samples collected from Zhejiang University Cancer Institute (ZUCI)

A tissue microarray (TMA) containing 110 RCC samples collected from ZUCI was used for IHC staining to evaluate HOXC6 expression. This experiment was approved by the Ethics Committee of the Second Affiliated Hospital, School of Medicine, Zhejiang University. A primary antibody against HOXC6 (1:50, Abcam, ab41587) was used for IHC staining. The HOXC6 expression score was blindly evaluated independently by two pathologists using an immunoreactivity score (IRS) based on the percentage of positive cells and the intensity of staining. In cases when the two pathologists provided a different score, a third pathologist was asked to evaluate the slide. The percentage of positive cells was graded as follows: 0 (negative), 1 (<10%), 2 (10–50%), 3 (51–80%), and 4 (>80%). The intensity of staining was graded as follows: 0 (no color reaction), 1 (mild reaction), 2 (moderate reaction), and 3 (intense reaction). The IRS was the product of the two scores. In this study, HOXC6 expression was defined as low (IRS ≤ 6) or high (IRS ≥ 9). Ten samples with IRS = 8 were excluded, which led to 100 samples being retained for subsequent survival analysis (Table S1).

### Cell culture

The HT29, HCT116, SW620, RKO, and HEK 293T cell lines were purchased from ATCC and cultured with RPMI 1640 medium (Gibco) containing 10% fetal bovine serum (FBS, BI Industry). The cells were incubated at 37 °C with 5% CO_2_.

### Stable gene overexpression and knockdown using a lentiviral transfection system

HOXC6-overexpressed, HOXC6-shRNA, and negative control lentivirus were purchased from GeneChem (Shanghai, China). In short, overexpression plasmids were transfected into 293T cells together with the Lentivector Expression System to produce lentivirus. For infection, 10^5^ cells were plated into 6-well plates and cocultured with 2.5 × 106 transducing-units (TU) virus in the presence of 1X HitransG (GeneChem, Shanghai, China) and standard medium. Twelve to 15 h later, the medium was replaced with fresh complete culture medium. After 72 h of transfection, 2 mg/ml puromycin was added to the culture medium for HCT116 or RKO selection, respectively. Western blot and quantitative reverse transcription-polymerase chain reaction (qRT-PCR) were utilized to confirm HOXC6 overexpression and knockdown.

### siRNA knockdown

Cells were plated into 6-well plates. After the cells grew to 50–60% confluence, Lipo3000 (Invitrogen) with specific siRNA (Genepharma, Shanghai) was added to the cells according to the manufacturer’s suggestions. The final concentration of siRNA was 100 nM. Cells were incubated with siRNA for 48 h and then harvested for protein and RNA extraction. The sequences of β-catenin siRNA were as follows: sense: 5′-CCUUCACUCAAGAACAAGUTT-3′ and antisense: 5′-ACUUGUUCUUGAGUGAAGGTT-3′. The sequences of DKK1 siRNA were as follows: sense: 5′-CCCGGGUCUUUGUCGCGAUTT-3′ and antisense: 5′-AUCGCGACAAAGACCCGGGTT-3′. The sequences of HOXC6 siRNA were as follows: sense: 5′-GAAAGCCAGUAUCCAGAUUTT-3′ and antisense: 5′-AAUCUGGAUACUGGCUUUCTT-3′. The negative control siRNA sequences were as follows: sense: 5′-UUCUCCGAACGUGUCACGUTT-3′ and antisense: 5′-ACGUGACACGUUCGGAGAATT-3′.

### Transwell migration and invasion assays of tumor cells

Cell invasion and migration were examined by Transwell assays with or without Matrigel. Approximately 10^4^ cells were plated into the upper chamber with RPMI 1640 medium without FBS. RPMI 1640 medium supplemented with 20% FBS was added to the lower chamber. After 72 h of culture for HCT116 and RKO cells, the cells in the upper chamber were scarpered, and cells under the upper chamber were fixed with 4% formalin and stained with crystal violet. The migrated/invaded cells were counted by light microscopy, and the mean cell number of five random visual fields at a magnification of ×200 was recorded.

### Cell proliferation

Approximately 10^3^ cells were plated into 96-well plates. Cell viability was measured at 1, 3, 5, and 7 days after plating. Cell Counting Kit-8 (CCK-8, Dojindo, Japan, CK04) was utilized for cell viability testing. Cell culture medium was used as a blank control. After 2–3 h of incubation, an optimal density (OD) value of 450 nm was used to detect cell proliferation. Experiments were carried out in triplicate. 5-Fluorouracil (5-FU), irinotecan, and oxaliplatin were purchased from Selleck.

### Protein extraction and western blotting

RIPA buffer (Beyotime) with 1% protease inhibitor cocktail (Roche Applied Science) was used for total protein extraction. For cytoplasmic and nuclear protein fractionation, a Nuclear and Cytoplasmic Protein Extraction Kit (Thermo Scientific, Catalog Number: 78833) was utilized. Next, 10% SDS-polyacrylamide gel electrophoresis (SDS-PAGE) gels were used to separate proteins according to their molecular weights. Then, the proteins were transferred onto polyvinylidene fluoride (PVDF) membranes by electrophoresis. After blocking with 5% nonfat milk, the PVDF membranes were incubated with primary antibodies followed by horseradish peroxidase (HRP)-linked secondary antibodies. Enhanced chemiluminescence (ECL) reagent was used to detect the protein bands. The primary antibodies used for western blotting were as follows: anti-HOXC6 (Santa Cruz, sc-376330), anti-β-catenin (Abcam, ab32572), anti-DKK1 (Abcam, ab109416), anti-c-Jun (Cell Signaling Technology, #9165), anti-EMT antibody kit (Cell Signaling Technology, #9782), anti-RNF43 (Abcam, ab84125), anti-Axin2(CST, #5863S), anti-Histone H3 (Huabio, Hangzhou, M1306–4), and anti-β-tubulin (Huabio, Hangzhou, M1305–2). All the antibodies used in this study were detailed in Table S2.

### Co-IP assay

For the co-IP assay, HOXC6-OE and NC HCT116 cell lines were grown to 80–90% confluence in a 10 cm dish. Subsequent steps were performed using the FLAG Immunoprecipitation Kit (Sigma, FLAGIPT1) according to the manufacturer’s instructions with some minor adaptations. Finally, proteins that bound to the gels were eluted with the sample buffer in the kit and boiled for 3 min. The eluates were subjected to western blotting and liquid chromatography–mass spectrometry/mass spectrometry (LC-MS/MS) (oebiotech, Shanghai) identification.

### RNA isolation and qRT-PCR

Total RNA was extracted from HT29, HCT116, RKO, and SW620 cells using Trizol following a standard protocol. The Takara PrimeScript TM RT Master Mix Kit (Takara, RR036Q) was used for reverse transcription. The iTaq Universal SYBR Green Supermix (BioRad) and Applied Biosystems 7500 Fast Real-Time PCR System were applied for qRT-PCR. GAPDH was used as the loading control. Experiments were carried out in triplicate. The results were calculated as follows: ΔCT = CT _Experimental/NC_-CT_GAPDH_, ΔΔCT = ΔCT _Experimental/NC_-ΔCT _NC,_ foldchange=2^−ΔΔCT^. The primers used for qRT-PCR are as follows.

**Table:** 

	Forward sequence (5′ to 3′)	Reverse sequence (5′ to 3′)
HOXC6	GAGAATGTCGTGTTCAGTTC	GATCTGTCGCTCGGTCAGGCAA
GAPDH	ATCCCATCACCATCTTCCAG	TGAGTCCTTCCACGATACCA
MLH1	CTCTTCATCAACCATCGTCTGG	GCAAATAGGCTGCATACACTGTT
β-catenin	TCTGAGGACAAGCCACAAGATTACA	TGGGCACCAATATCAAGTCCAA
DKK1	TCACACCAAAGGACAAGAAGGT	GGACCAGAAGTGTCTAGCA

### Evaluation of DKK1 by enzyme-linked immunosorbent assay (ELISA)

Approximately 5×10^5^ cells were plated into 6-well plates. After the specified treatments according to the manufacturer’s suggestions, the culture medium was harvested simultaneously. Detection of secreted human DKK1 was performed using DKK1-specific ELISA kits (BOSTER, Wuhan, China, Lot: EK0867) according to the manufacturer’s instructions.

### Dual luciferase reporter assay

The 2 kb sequence of the *DKK1* promoter (retrieved from the Ensembl database, www.ensembl.org) containing the predicted HOXC6 recognition motif (TATTTAAT, described previously^17,18^) was amplified by PCR using genomic DNA from HCT116 cells (forward sequence 5′ to 3′ CAGCTAGCACTTTCTCTTCTTTTGCCCCAGG, reverse sequence 5′ to 3′ AGAGCTCCTGACTGCAGGGAGCACAGA). The PCR product was then cloned into the pGL3 control vector (Invitrogen) by using the NheI and SacI restriction sites. A mutant of the predicted HOXC6 recognition motif (CGAACCAG) within the *DKK1* promoter was synthesized using a fast site-directed mutagenesis kit (TIANGEN, KM101). The HOXC6 sequence was amplified by PCR using complementary DNA of total RNA extracted from HCT116 cells (forward sequence 5′ to 3′ ATGCAGCTAGCATGAATTCCTACTTCACTAACCCTTCCT, reverse sequence 5′ to 3′ TCGAAGCTTTCACTCTTTCTGCTTCTCCTCTTCTGT). The PCR product was then cloned into the pcDNA3.1 (-) vector (Invitrogen) at the NheI and HindIII restriction sites.

For the luciferase activity detection assay, HEK293T cells were co-transfected with a vector containing the wild-type (WT) or mutant promoter of DKK1 and the pcDNA3.1 (-) vector containing HOXC6 or the pcDNA3.1 (-) vector control and cultured. After 48 h, the dual luciferase activities were examined using the Dual-Luciferase Reporter Assay System (Promega, Cat. #E1910).

### HOXC6 and DKK1 fluorescence colocalization experiment

HOXC6 and DKK1 expression plasmid were purchased from Genomeditech (Shanghai, China). H_HOXC6 (3223, NM_004503.4) was cloned into PGMLV-CMV-MCS-3×Flag-EF1-mScarlet-T2A-Blasticidin vector and H_DKK1 (22943, NM_012242.4) was cloned into PGMLV-CMV-MCS-EF1-ZsGreen1-T2A-Puro vector. Both plasmids were transfected into 293T or HCT116 cells using Lipo3000 (Invitrogen) according to recommended protocol, 4% paraformaldehyde was used to fix cells after 48 h cultivation. After adding DAPI, a confocal microscope (Zeiss) was used for taking photographs at 400X resolution.

### Animal experiments

The animal experiments were approved by Zhejiang University and conducted according to the Animal Study Guidelines of Zhejiang University. Five-week-old female nude mice (BALB/c) were used for the animal study. The metastatic tumor mouse model was constructed by tail vein injection of 2 × 10^6^ HCT116 tumor cells. The mice were randomly divided into two groups: control (*n* = 5), and HOXC6-OE (*n* = 5). One mouse in each group died immediately after the injection of tumor cells because of pulmonary embolism. The mice were sacrificed 46 days later, and the lungs and livers of the mice were dissected and fixed in 10% buffered formalin for hematoxylin-eosin (HE) staining. The subcutaneous tumor formation mouse model was constructed by injection of 10^7^ HCT116 tumor cells. The mice were randomly divided into three groups: control (*n* = 5), HOXC6-KD (*n* = 5), and HOXC6-OE (*n* = 5). The mice were sacrificed 21 days later.

### Statistical analysis

All statistical analyses and graphical representations were performed in the R programming language (×64, version 3.5.1) and GraphPad Prism7 unless otherwise specified.

## Results

### HOXC6 was overexpressed in RCC and associated with poor prognosis

**Fig. 1:**
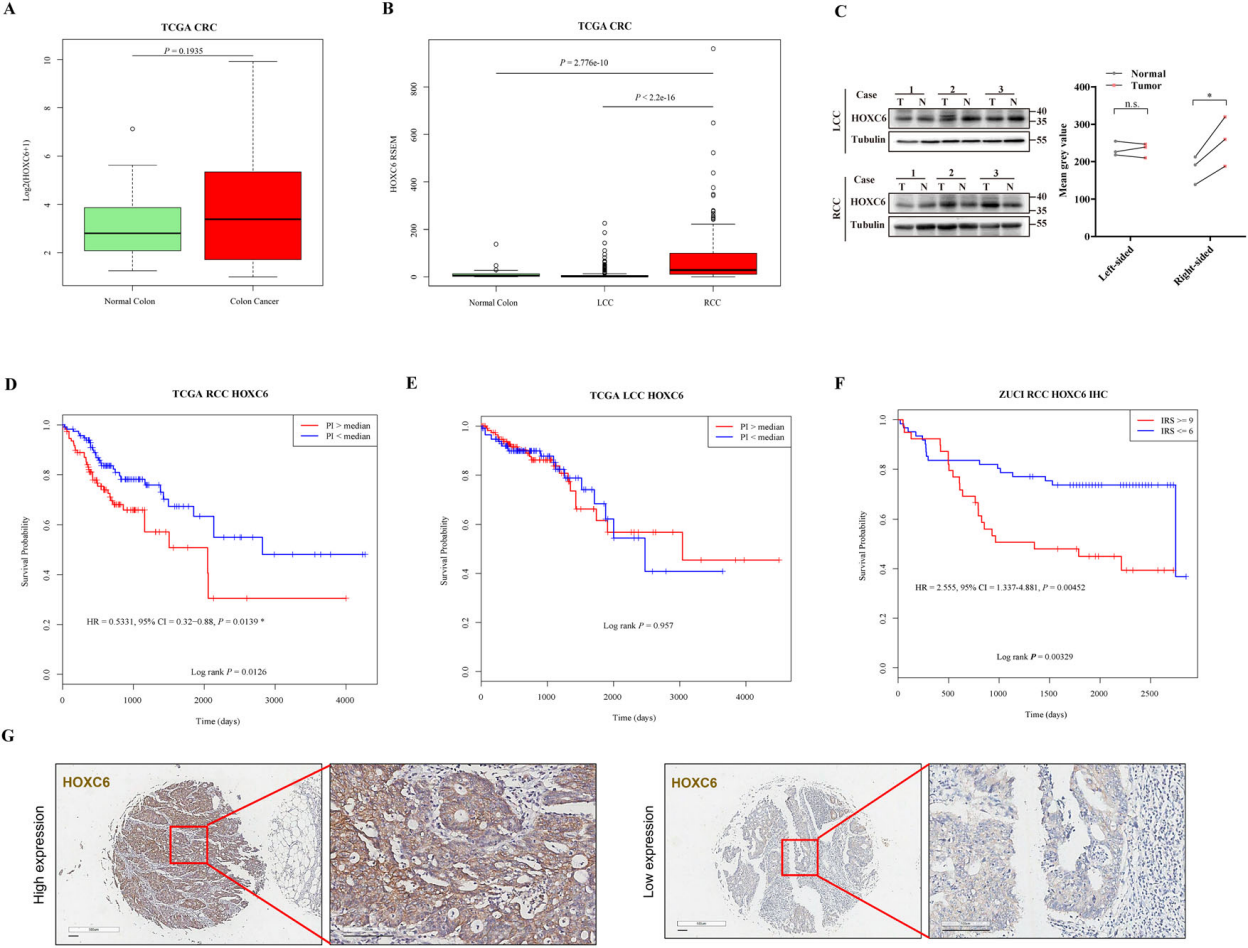
Expression of HOXC6 and prognostic value in human CRC. **A** No difference in HOXC6 expression was observed between CRC and normal tissues by using TCGA samples. **B** HOXC6 was overexpressed in RCC but not in LCC compared to normal colon tissues based on TCGA samples. **C** Left: Western blot of fresh-frozen tissues from RCC and LCC showed that HOXC6 was overexpressed in RCC but not in LCC compared to paired adjacent normal colon tissues. Right: Adjusted mean gray values by control gray values of WB. **D**. KM plot showing that HOXC6 high-expression group had poor prognosis compared to the low-expression group in RCC. **E** The KM plot showed that there was no prognostic difference between the HOXC6 high-expression and low-expression groups in LCC. **F** KM plot showing that the HOXC6 high-expression group had a poor prognosis compared to the low-expression group in the ZUCI RCC samples (*n* = 100). **G** Representative images of the HOXC6 high-expression and low-expression groups. Scale bars indicate 100 µm. *: *P* < 0.05.

To explore the expression pattern of HOXC6 in CRC, we first compared the overall expression levels between CRC and adjacent normal tissues based on TCGA samples. The results revealed that there was no significant difference between CRC and normal colon tissues (*P* = 0.2, Wilcoxon test, Fig. 1A). Then, CRC was further divided into RCC (including cecum, ascending colon, hepatic flexure, and transverse colon) and LCC (including splenic flexure, descending colon and sigmoid colon). Intriguingly, the expression level of HOXC6 was significantly elevated in RCC compared to LCC (*P* < 2.2E−16, Wilcoxon test) and normal colon tissues (*P* = 2.776E−10, Wilcoxon test, Fig. 1B). To further validate the overexpression of HOXC6 in RCC, western blotting was performed in six pairs of fresh-frozen CRC and adjacent normal tissue samples collected at ZUCI. The results showed that HOXC6 was significantly upregulated in RCC compared to adjacent normal tissue but not in the paired LCC tissue (*P* = 0.04, paired *t-*test, Fig. 1C), which was consistent with TCGA observations.

Next, we assessed the prognostic value of HOXC6 based on TCGA samples and found that high expression of HOXC6 was significantly associated with poor clinical outcome in RCC (log rank *P* = 0.0126, Fig. 1D). The median overall survival (OS) times for HOXC6- and HOXC6+ groups were 2821 days (95% CI, 2134 to NA) and 2047 days (95% CI, 1158 to NA), respectively. However, this association was not held in LCC (log rank *P* = 0.957, Fig. 1E). To validate the prognostic role of HOXC6 in RCC independently, a TMA of 100 RCCs collected from the ZUCI cohort was used for the IHC evaluation. The KM plot showed that high expression of HOXC6 was significantly correlated with poor OS compared with the HOXC6 low-expression group in RCC (log rank *P* = 0.004, Fig. 1F, G), which was consistent with the TCGA results. The median OS time of HOXC6- and HOXC6+ groups were 2749 days (95% CI, 2749 to NA) and 1349 days (95% CI, 795 to NA), respectively.

### High expression of HOXC6 promotes migration, invasion, and metastasis of RCC

**Fig. 2:**
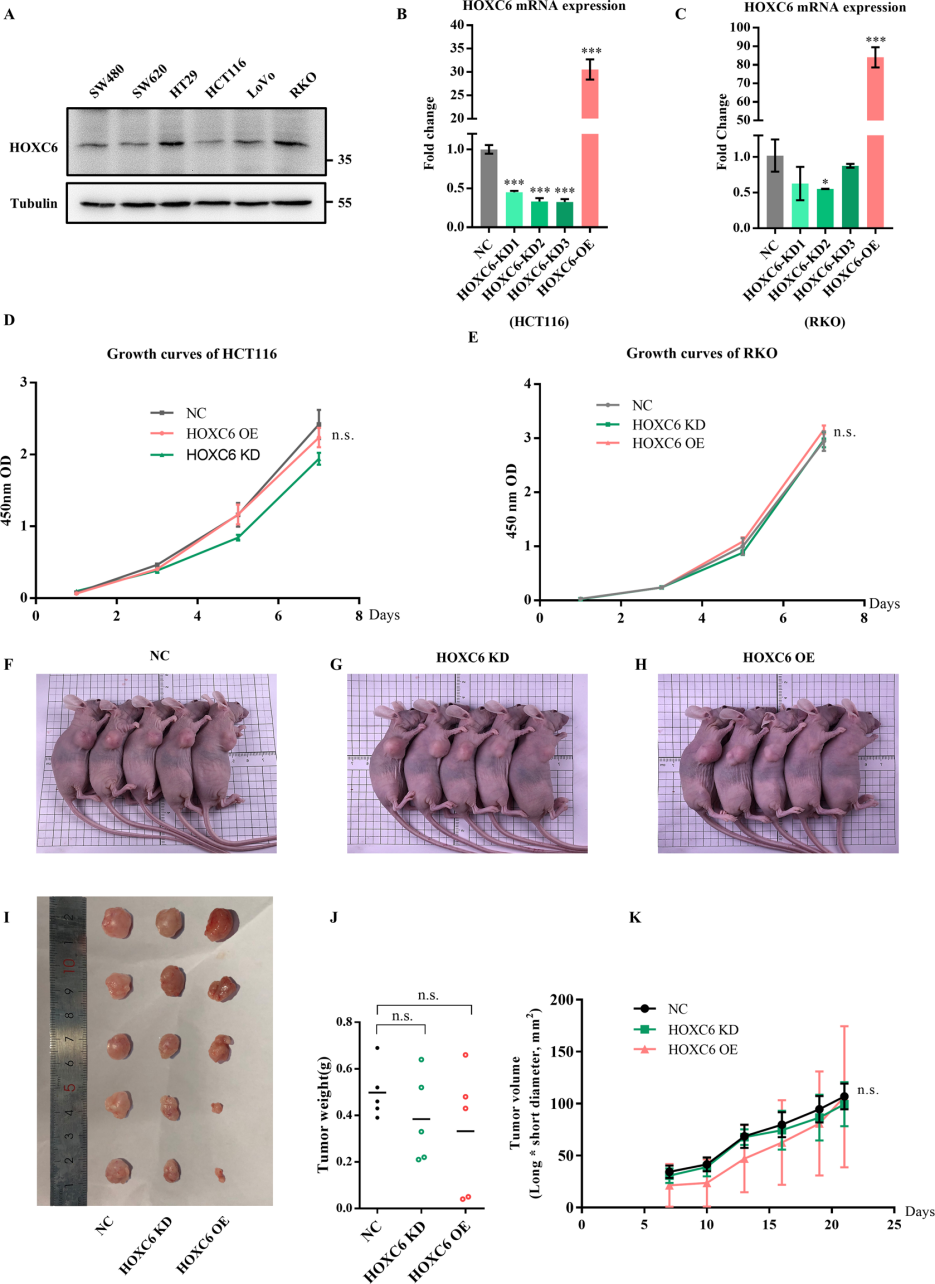
HOXC6 did not alter proliferation ability of HCT116 and HT29 cells in vitro and in vivo. **A** Expression of HOXC6 in six CRC cell lines. **B** Identification of stable HOXC6 overexpression and knockdown in HCT116 cell lines using qRT-PCR. **C** Identification of stable HOXC6 overexpression and knockdown in RKO cell lines using qRT-PCR. **D** Growth curves of HCT116 cells in the NC, HOXC6-OE and HOXC6-KD groups. **E** Growth curves of RKO cells in the NC, HOXC6-OE and HOXC6-KD groups. **F** The subcutaneous NC HCT116 tumor cell injection mice. **G** The subcutaneous HOXC6-KD HCT116 tumor cell injection mice. **H** The subcutaneous HOXC6-OE HCT116 tumor cell injection mice. **I** The tumors dissected from NC, HOXC6-KD and HOXC6-OE mice. **J** The tumor wight for dissected tumors from NC, HOXC6-KD and HOXC6-OE mice groups. **K** The tumor growth curve evaluated by tumor long*short diameter for NC, HOXC6-KD and HOXC6-OE groups. *P < 0.05; ***P < 0.001; n.s.: no significance.

To determine the reason why the high expression of HOXC6 correlates with an unfavorable outcome in RCC, we first compared the clinical characteristics between the HOXC6- and HOXC6+ groups by using TCGA samples. Interestingly, we found that approximately two-fold more samples (~15%) had distant metastasis in the HOXC6+ group (Table S1). To verify the metastatic activity of HOXC6, lentivirus methods were used to construct HOXC6 overexpression (HOXC6-OE) and knockdown (HOXC6-KD) cell lines in HCT116 and RKO cells because the expression of HOXC6 was high in RKO cells and low in HCT116 cells based on testing six common CRC cell lines (Fig. 2A–C). Then, tumor proliferation was evaluated in vitro and in vivo. Notably, no significant change in proliferation after upregulation or downregulation of HOXC6 in HCT116 and RKO cell lines was observed (Fig. 2D, E). An in vivo tumor formation mouse model by subcutaneous injection of stable HOXC6-OE and HOXC6-KD HCT116 cells demonstrated HOXC6 didn’t change tumor cell proliferation (Fig. 2F–K), which was in concordance with in vitro results.

**Fig. 3:**
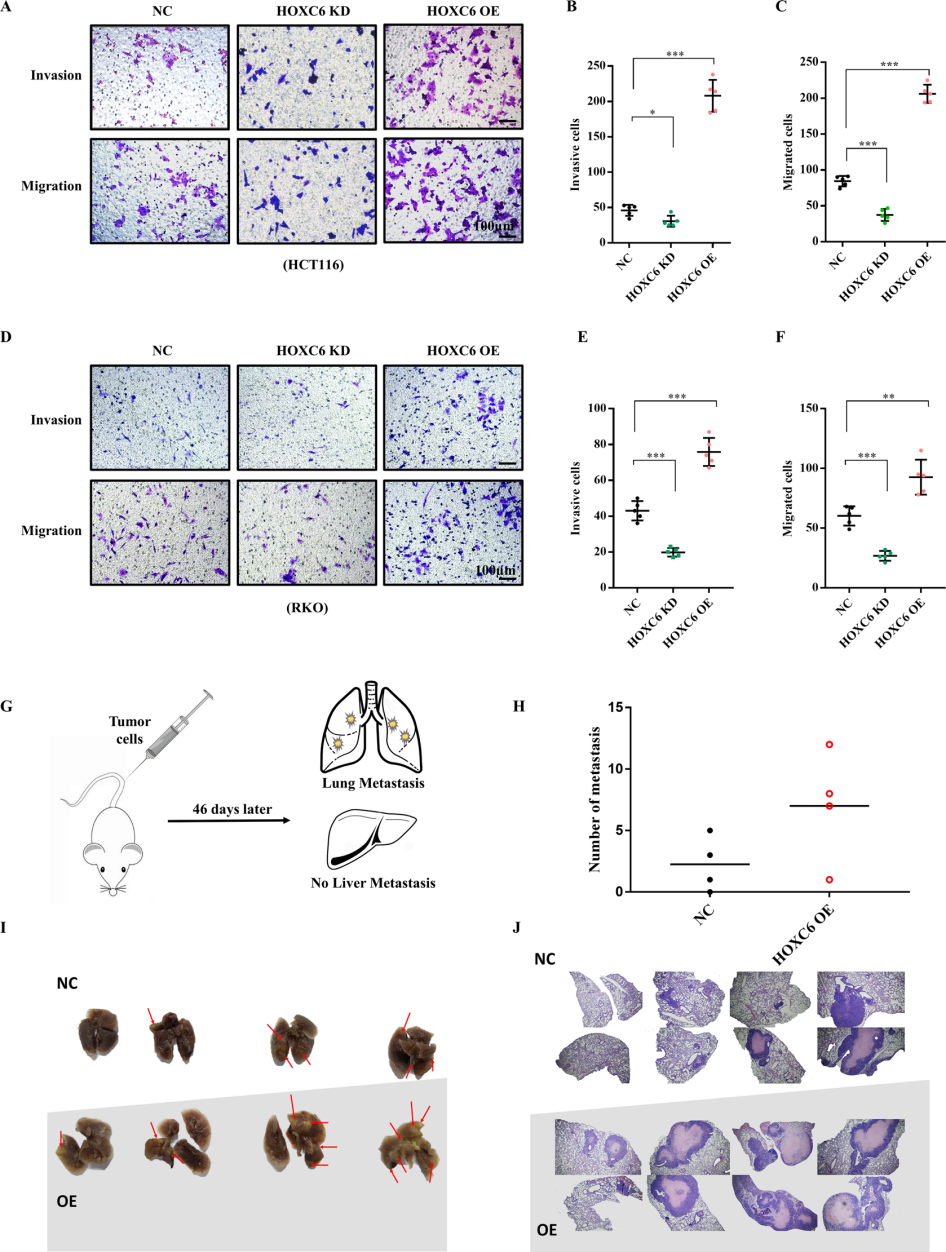
Overexpression of HOXC6 promotes the migration and invasion of CRC cells in vitro and in vivo. **A** Transwell experiments with and without matrigel with HCT116 cells in the NC HOXC6-KD and HOXC6-OE groups. Scale bars indicate 100 µm. **B** Numbers of invading HCT116 cells in the NC, HOXC6-KD, and HOXC6-OE groups. **C** Numbers of migrating HCT116 cells in NC, HOXC6-KD, and HOXC6-OE groups. **D** Transwell experiments with and without matrigel of RKO cells in the NC HOXC6-KD and HOXC6-OE groups. **E** Numbers of invading RKO cells in the NC HOXC6-KD and HOXC6-OE groups. **F** Numbers of migration RKO cells in the NC HOXC6-KD and HOXC6-OE groups. **G** Schematic diagram of a metastatic tumor model of nude mice with tumor cells injected into the tail vein. **H** The number of metastatic foci in mouse lungs in the NC and HOXC6-OE HCT116 groups. **I** Images of isolated mouse lungs in the NC and HOXC6-OE HCT116 groups. **J** HE staining images of mouse lungs in the NC and HOXC6-OE HCT116 groups. *: *P* < 0.05; **: *P* < 0.01; ***: *P* < 0.001. n.s.: no significance.

Then, tumor invasion, migration, and metastasis were evaluated in vitro and in vivo. Interestingly, we found that the invasion and migration ability were significantly enhanced in the HOXC6-OE group and significantly hampered when HOXC6 was downregulated in both HCT116 (Fig. 3A–C) and RKO cell lines (Fig. 3D–F). An in vivo metastatic mouse model by tail vein injection of stable HOXC6-overexpressing HCT116 cells demonstrated metastases in the lungs (Fig. 3G), and the number of metastases was larger in the HOXC6-OE group than in the NC group (Fig. 3H, I). HE staining showed that the size of metastatic foci was also larger in the HOXC6-OE group (Fig. 3J).

### MLH1 silencing was related to enhancement of HOXC6

To disentangle the reason why HOXC6 was much higher expressed in RCC than LCC, pairwise correlation coefficients of HOXC6 and all the other detected 20,501 genes in TCGA were calculated because functionally closely related gene pairs may exhibit similar expression patterns either in positive or negative trends. Of note, we found that the expression of *MLH1* was significantly negatively correlated with HOXC6 expression (*P* = 1.4E−09, Pearson correlation *r* = −0.37, Fig. S1A). To validate the potential regulatory relationship, *MLH1* was knocked down by customized siRNA, and HOXC6 was found to be increased at 1.3-fold in HT29 cells (*P* < 0.05, *t*-test, Fig. S1B) and 1.7-fold in SW620 cells (*P* < 0.001, *t*-test, Fig. S1C). This evidence indicated that inhibition of MLH1 may partially explains why HOXC6 was more highly expressed in RCC than LCC.

### HOXC6 contributed to activation of Wnt/β-catenin pathway and EMT

**Fig. 4:**
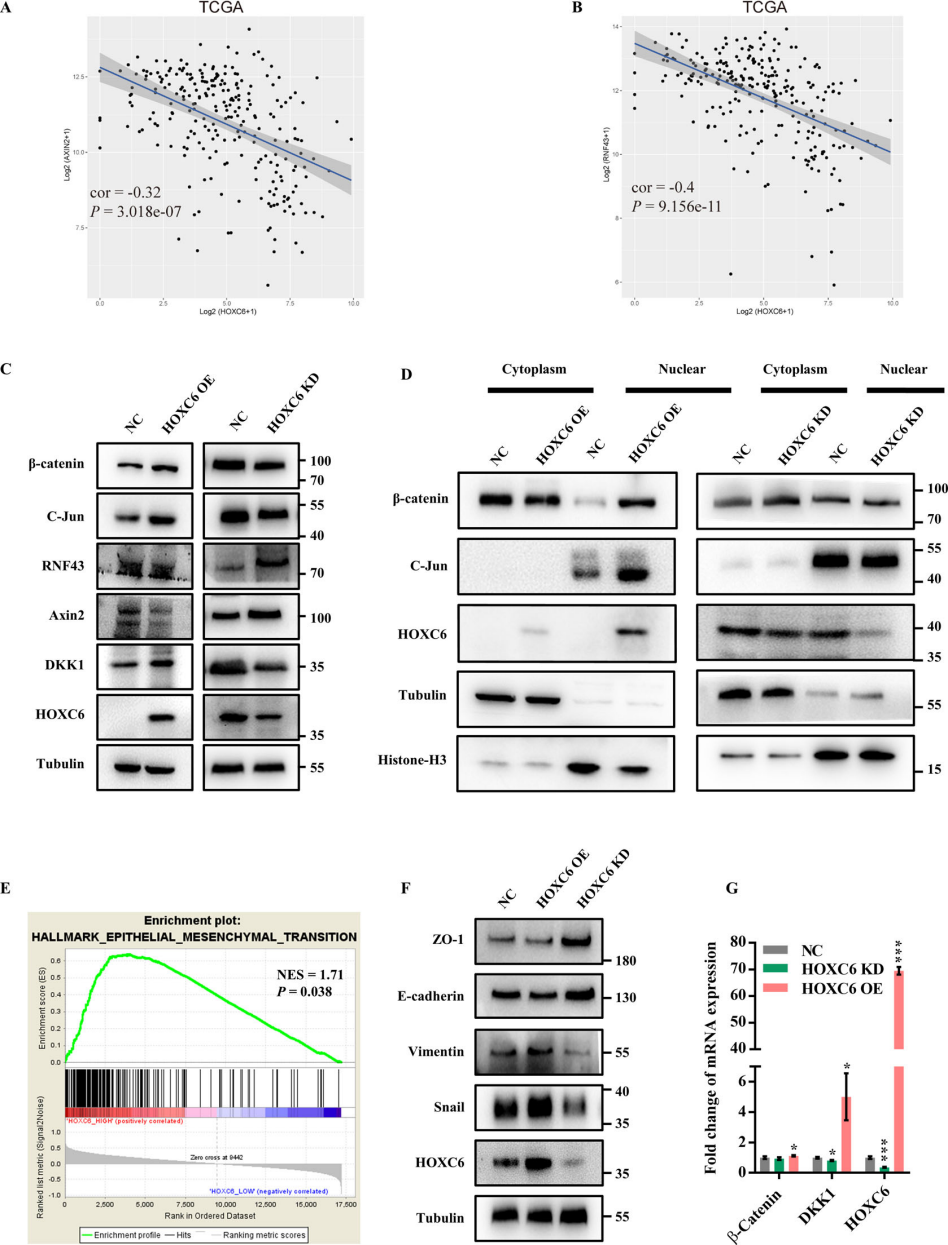
HOXC6 contributes to activation of Wnt/β-catenin pathway and EMT. **A** Pairwise correlation between Axin2 and HOXC6 using TCGA expression profiles. **B** Pairwise correlation between RNF43 and HOXC6 using TCGA expression profiles. **C** Expression of β-catenin, c-Jun, RNF43, Axin2, DKK1, and HOXC6 was evaluated by WB in whole-cell lysates of HOXC6-OE, HOXC6-KD, and their control groups in the HCT116 cell line. **D** The expression of β-catenin, c-Jun, and HOXC6 was evaluated in the cytoplasm and nucleus of HCT116 cells in the HOXC6-OE, HOXC6-KD, and control groups. Tubulin was used as a cell plasma protein and whole-cell protein loading control. Histone-H3 was used as a nuclear protein loading control. **E** GSEA showed that the EMT process was significantly enriched in the HOXC6-overexpressing group using TCGA dataset. **F** Protein expression of ZO-1, E-cadherin, vimentin, snail, and HOXC6 in three different treated HCT116 groups. **G** Relative mRNA expression of β-catenin, DKK1, and HOXC6 in the NC, HOXC6-KD, and HOXC6-OE groups in HCT116 cells. *: *P* < 0.05; ***: *P* < 0.001.

A detailed mechanism of how HOXC6 promotes metastasis in tumor cells may elucidate the association between high expression of HOXC6 and RCC aggressiveness. We first performed RNAseq of the HOXC6-OE group and NC group. DKK1, a known secretory Wnt/β-catenin inhibitor^19^, was upregulated 13.8-fold in HOXC6-OE cells (Table S3). Then, Co-IP and subsequent LC-MS/MS were used to discover the HOXC6 binding partners. Intriguingly, we found that DKK1 could bind to HOXC6 (score 23, Mascot search result, Table S4). Furthermore, a significant negative correlation was observed between HOXC6 and Axin2/RNF43 (Fig. 4A, B), and they were the top significant DEGs under high expression of HOXC6 (Table S5). RNF43 and Axin2 are inhibitors of the WNT pathway and accelerate the degradation of β-catenin in the cytoplasm. These results indicated that HOXC6 was closely related to activation of the Wnt/β-catenin pathway in RCC. To further disentangle the relationship between HOXC6 and the Wnt/β-catenin pathway, we first inspected the changes in expression of canonical members of the Wnt pathway. Notably, total β-catenin and nucleus-derived β-catenin were elevated in the HOXC6-OE group and reduced in the HOXC6-KD group (Fig. 4C, D). The expression of c-Jun, a known target of the Wnt/β-catenin pathway, was consistent with the expression trend of β-catenin (Fig. 4C, D). In addition, RNF43 and Axin2 exhibited a negative expression trend with β-catenin (Fig. 4C), which was consistent with TCGA observations. These results suggested that overexpression of HOXC6 was related to activation of the Wnt/β-catenin signaling pathway in RCC.

To better understand the molecular pathways associated with high HOXC6 expression, GSEA was performed to identify prominent signatures. Interestingly, EMT (NES = 1.71, *P* = 0.038, Fig. 4E) was characteristic of HOXC6 overexpression. This signature was confirmed by the overexpression of HOXC6 in HCT116 cells by upregulating vimentin and snail (Fig. 4F). Mesenchymal–epithelial transition (MET) was accomplished by knockdown of HOXC6 in HCT116 cells (Fig. 4F), indicating that HOXC6 plays a critical role in EMT and MET of RCC.

### HOXC6 activated Wnt/β-catenin pathway via inhibiting DKK1 excretion

**Fig. 5:**
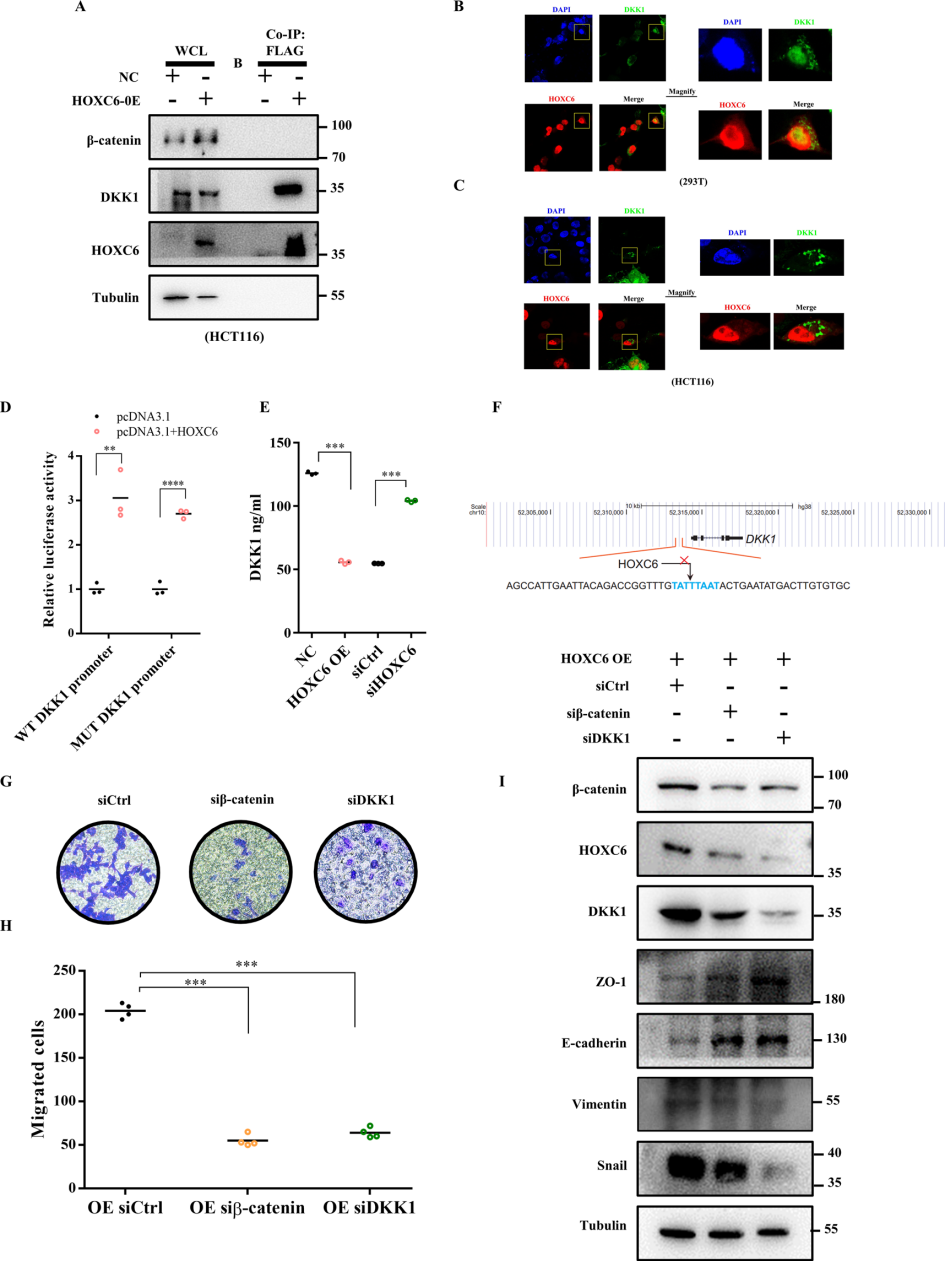
HOXC6 activates Wnt/β-catenin via inhibiting DKK1 excretion. **A** Co-IP using Flag-gel was performed with HCT116 cells in the HOXC6-OE and NC groups. Whole-cell lysate was loaded as a positive control. β-catenin, DKK1, and HOXC6 were evaluated in the Flag-HOXC6 complex. **B** HOXC6 and DKK1 fluorescent colocalization in 293 T cells. **C** HOXC6 and DKK1 fluorescent colocalization in HCT116 cells. **D** Dual luciferase assay testing the ability of HOXC6 to bind to the DKK1 promoter with WT and mutant sequences in HEK 293T cells. **E** ELISA detection of DKK1 concentration in cell supernatants in different HCT116 experimental groups. **F** Schematic diagram for the potential binding sequence of HOXC6in the DKK1 promoter. **G** Transwell experiment in the HOXC6-OE siCtrl, HOXC6-OE siβ-catenin, and HOXC6-OE siDKK1 groups in HCT116 cells. **H** Numbers of migration HCT116 cells in the HOXC6-OE siCtrl, HOXC6-OE siβ-catenin, and HOXC6-OE siDKK1 groups. **I** Expression of β-catenin, HOXC6, DKK1, ZO1, E-cadherin, Vimentin, and snail in HOXC6-OE siCtrl, HOXC6-OE siβ-catenin, and HOXC6-OE siDKK1 groups in HCT116 cells. ***: *P* < 0.001.

Given that DKK1 is a secretory Wnt/β-catenin inhibitor and upregulated by HOXC6, a question remains of why HOXC6 promotes DKK1 expression and upregulation of DKK1 could led to activation of Wnt/β-catenin pathway. To answer this contradiction, we first validated the high-throughput RNA sequencing results by qRT-PCR and WB. Indeed, the expression of DKK1 was significantly upregulated in the HOXC6-OE group and decreased in the HOXC6-KD group at both the mRNA and protein levels (*P* < 0.05, *t*-test, Fig. 4C, G). Co-IP and subsequent WB experiments showed that DKK1 was able to bind to HOXC6, but β-catenin was not (Fig. 5A). To rule out the possibility of post-lysis interaction of DKK1 and HOXC6, we also performed the fluorescence co-localization experiment and found that a part of HOXC6 and DKK1 were co-localized in both 293 T cells and HCT116 cells, which supported that HOXC6 could directly bind with DKK1 (Fig. 5B, C). Since HOXC6 is a transcription factor, the dual luciferase reporter assay was performed to explore whether HOXC6 directly upregulated DKK1 by binding to its promoter. However, there was no direct relationship between HOXC6 and DKK1 (Fig. 5D). We hypothesized that loss of DKK1 function could be caused by inhibiting its secretion despite high intracellular expression. As expected, upregulation of HOXC6 significantly inhibited DKK1 excretion and downregulation of HOXC6 increased DKK1 extracellular secretion (*P* < 0.001, *t*-test, Fig. 5E and Fig. S1D). In brief, upregulation of DKK1 in the cytoplasm could still activate the Wnt/β-catenin pathway through inhibition of DKK1 secretion by more HOXC6 binding but not by its transcriptive effect although a putative HOXC6 binding motif (-443 bp upstream of the transcription start site) was found within the DKK1 promoter (Fig. 5F).

Next, we evaluated whether HOXC6 promotes EMT through the DKK1/Wnt/β-catenin signaling cascade because EMT is closely associated with metastasis. Transwell experiments showed that knockdown of β-catenin or DKK1 significantly inhibited the migration of HCT116 cells (*P* < 0.001, *t*-test, Fig. 5G, H). Moreover, knockdown of β-catenin or DKK1 in the stable HOXC6-OE HCT116 cell line showed that EMT could be reversed by upregulating ZO-1 and E-cadherin and downregulating vimentin and snail (Fig. 5I). HOXC6 and DKK1 were reduced by knockdown of β-catenin, suggesting that HOXC6 and DKK1 were target genes of the Wnt/β-catenin pathway (Fig. 5I). Somewhat strangely, both β-catenin and HOXC6 were reduced if DKK1 was downregulated, implying that a subtle negative feedback regulatory mechanism was present in RCC. Collectively, these results revealed that overexpression of HOXC6 was closely associated with EMT via activation of the Wnt/β-catenin pathway.

### Silencing of HOXC6 is a promising strategy for RCC treatment

**Fig. 6:**
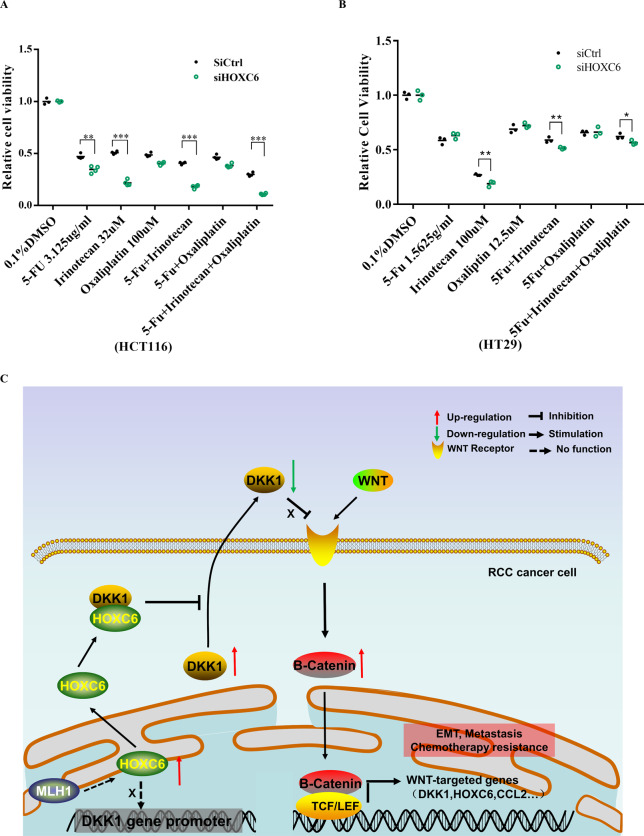
Inhibition of HOXC6 contributes to enhanced chemotherapy sensitivity. **A** HCT116 cells in the siHOXC6 group were more sensitive to 5-Fu, irinotecan, 5-Fu + irinitecan and 5-Fu + irinitecan+oxaliplatin compared to those in the siCtrl group. **B** HT29 cells in the siHOXC6 group were more sensitive to irinotecan, 5-Fu + irinitecan and 5-Fu + irinitecan+oxaliplatin compared to those in the siCtrl group. **C** Schematic diagram of HOXC6 promote metastasis via DKK1/Wnt/β-catenin axis in RCC. *: *P* < 0.05; **: *P* < 0.01; ***: *P* < 0.001.

In view of the close association of EMT and HOXC6, we next assessed whether knockdown of HOXC6 would be a feasible therapeutic strategy for RCC treatment. First, to test the effect of HOXC6 on drug sensitivity, 5-FU, irinotecan, and oxaliplatin, which are the most frequently used chemotherapeutic agents in CRC clinical practice, were used to assess the target possibilities. The 48-h IC50 values for 5-FU, irinotecan, and oxaliplatin were 3.125 µg/ml, 32, and 100 µM in HCT116 cells (Fig. S1E–G) and 1.5625, 100, and 12.5 µM in HT29 cells (Fig. S1H–J), respectively. Drug susceptibility testing showed that knockdown of HOXC6 made HCT116 cells more sensitive to 5-FU (*P* < 0.01, *t*-test), irinotecan (*P* < 0.001, *t*-test), and the combinations of 5-FU + irinotecan and 5-FU + irinotecan+oxaliplatin (*P* < 0.001, *t*-test, Fig. 6A). In the HT29 cell line, a similar result was observed that tumor cells were more sensitive to irinotecan (*P* < 0.01, *t*-test), 5-FU + irinotecan (*P* < 0.01, *t*-test) and 5-FU + irinotecan+oxaliplatin (*P* < 0.05, *t*-test) by knockdown of HOXC6 (Fig. 6B).

## Discussion

Since RCC has a poorer prognosis than LCC, there is an urgent need to find molecular differences between these two subtypes. Given that canonical driver mutation genes such as *APC*, *TP53*, and *KRAS* are shared by LCC and RCC, it is tempting to believe that activation of specific oncogenes and/or silencing of tumor suppressors may better explain their prognostic difference and also provide clues for treatment. Recently, HOXB13, another member of the homeobox gene family, was found to be downregulated in RCC via hypermethylation and to play a tumor suppressive role in RCC^20^. However, HOXC6 was confirmed to be overexpressed in RCC and associated with poor prognosis in this study, which is in line with previous studies in other cancer types^11,21,22^.

With regard to the downstream regulatory cascade of HOXC6 participating in CRC progression, we found that upregulation of HOXC6 could induce EMT and mainly activate the Wnt/β-catenin pathway. Translocation of β-catenin from the cytoplasm to the nucleus, a sign of Wnt signaling activation, was enriched in the HOXC6-OE group and weakened in the HOXC6-KD group (Fig. 4D). Moreover, the two Wnt inhibitors, RNF43 and Axin2, had the opposite expression patterns compared to HOXC6. It’s widely known that EMT plays a very important role in the process of tumor metastasis and is characterized by decreased expression of epithelial markers, increased expression of interstitial markers and changes in cell morphology^23^. In CRC, it’s known that colon adenoma was initiated by aberrant Wnt/β-catenin signaling following loss of the tumor suppressor adenomatous polyposis coli (APC)^24^. Unfortunately, the Wnt/β-catenin signaling pathway lacks druggable targets currently, which hampered the clinical treatment. The activation of Wnt/β-catenin by HOXC6 provided potential therapeutic option for targeting upstream of Wnt/β-catenin pathway to inhibit tumor metastasis.

Given that DKK1 is a secreted inhibitor of the Wnt/β-catenin pathway^19^, HOXC6 could trigger the Wnt/β-catenin pathway to be continuously activated by inhibiting DKK1 secretion though DKK1 was upregulated by HOXC6 (Fig. 5E). Loss of HOXC6 has been shown to lead to DKK1 decrease in mouse pancreatic endocrine cells^25^. Considering the *DKK1* promoter has a HOXC6 putative binding motif, we further examined whether HOXC6 upregulates DKK1 directly. However, HOXC6 was not a direct upstream regulator of DKK1 but may indirectly upregulate DKK1 through β-catenin^26^. Furthermore, besides *DKK1*, *HOXC6* may also be a target gene of the Wnt/β-catenin pathway because the expression of DKK1 and HOXC6 was downregulated by β-catenin knockdown in HOXC6-OE HCT116 cells (Fig. 5I). Overall, the HOXC6/DKK1/Wnt/β-catenin axis contributes to the EMT process in RCC.

Last, we first found that downregulation of HOXC6 made HCT116 and HT29 cell lines more sensitive to irinotecan. Though irinotecan is widely used for advanced CRC in clinical practice, only 30–55% CRC patients benefit from irinotecan treatment^27^. UGT1A1 genotype can be used to guide irinotecan medication^28^. In this study, we extended the scope of irinotecan in CRC treatment but warrants further investigation.

## Conclusion

In this study, we found that HOXC6 was significantly overexpressed in RCC and associated with poor outcomes. High expression of HOXC6 promoted metastasis of RCC via activating the WNT pathway. Consequently, HOXC6 could be served as a metastatic predicting biomarker for early stage RCC and a promising drug target which warrants further investigation.

## Supplementary information

Figure S1

Table S1

Table S2

Table S3

Table S4

Table S5
