# Supplementary figures and images for: HomeoboxC6 promotes metastasis by orchestrating the DKK1/Wnt/β-catenin axis in right-sided colon cancer
Source: Cell Death Dis. 2021 Apr 1;12(4):337. doi: 10.1038/s41419-021-03630-x (PMC8016886; doi:10.1038/s41419-021-03630-x)

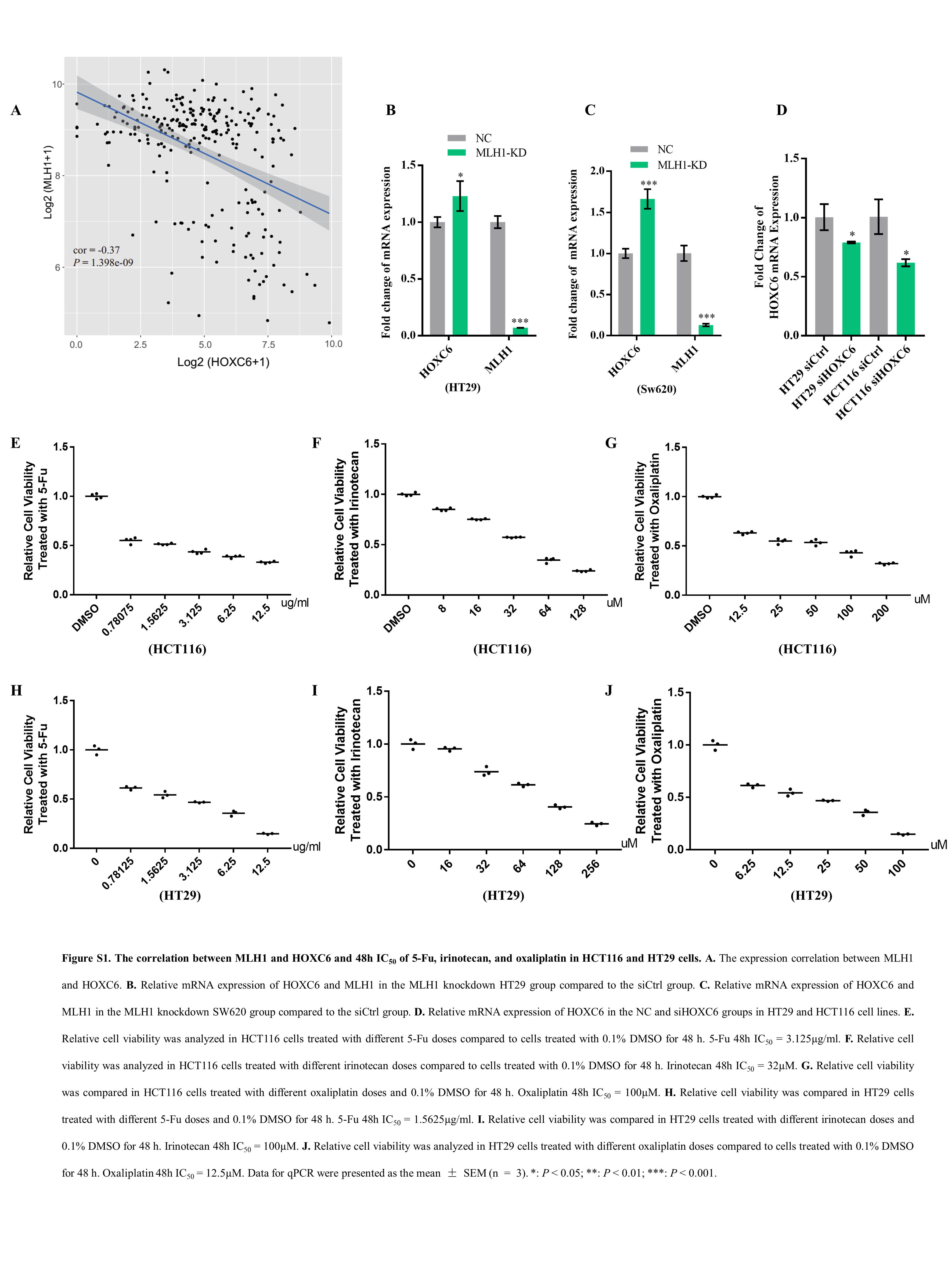

Supplement: Supplementary file 1 — Figure S1 [file 41419_2021_3630_MOESM1_ESM.tif]
