# Supplementary material for: HomeoboxC6 promotes metastasis by orchestrating the DKK1/Wnt/β-catenin axis in right-sided colon cancer
Source: Cell Death Dis. 2021 Apr 1;12(4):337. doi: 10.1038/s41419-021-03630-x (PMC8016886; doi:10.1038/s41419-021-03630-x)
Supplement: Supplementary file 2 — Table S1 [file 41419_2021_3630_MOESM2_ESM.docx]

| Table S1. Clinical characteristics of right-sided colon cancer patients. | | | | | | |
| --- | --- | --- | --- | --- | --- | --- |
|  | ZUCI | | | TCGA | | |
|  | **Low (IRS ≤6) (*n*=61)** | **High (IRS ≥9) (*n*=39)** | ***P*-value** | **Low (< Median) (*n*=121)** | **High (>Median) (*n*=120)** | ***P*-value** |
| **Gender** |  |  | 0.736 |  |  | 0.274 |
| Male | 35 | 20 |  | 70 | 60 |  |
| Female | 26 | 19 |  | 51 | 60 |  |
| **Age** |  |  | 0.837 |  |  | 0.425 |
| >60 | 26 | 16 |  | 25 | 31 |  |
| ≤60 | 34 | 24 |  | 96 | 89 |  |
| **Stage** |  |  | 0.52 |  |  | 0.085 |
| I | 1 | 1 |  | 28 | 15 |  |
| II | 31 | 20 |  | 51 | 52 |  |
| III | 21 | 13 |  | 28 | 33 |  |
| IV | 8 | 5 |  | 10 | 18 |  |
| unknown | 0 | 0 |  | 4 | 2 |  |
