# Supplementary material for: HomeoboxC6 promotes metastasis by orchestrating the DKK1/Wnt/β-catenin axis in right-sided colon cancer
Source: Cell Death Dis. 2021 Apr 1;12(4):337. doi: 10.1038/s41419-021-03630-x (PMC8016886; doi:10.1038/s41419-021-03630-x)
Supplement: Supplementary file 3 — Table S2 [file 41419_2021_3630_MOESM3_ESM.docx]

| **Antibody** | **Supplier** | **Catalogue Number** |
| --- | --- | --- |
| HOXC6-IHC | Abcam | ab41587 |
| HOXC6-WB | Santa Cruz | sc-376330 |
| β-catenin | Abcam | ab32572 |
| DKK1 | Abcam | ab109416 |
| c-Jun | CST | #9165 |
| EMT antibody kit | CST | #9782 |
| RNF43 | Abcam | ab84125 |
| Axin2 | CST | #5863S |
| Histone H3 | Huabio, Hangzhou | M1306-4 |
| β-tubulin | Huabio, Hangzhou | M1305-2 |

Table S2. List of antibodies used in this study.
